# One-Year Clinical Performance of Activa™ Bioactive-Restorative Composite in Primary Molars

**DOI:** 10.3390/children9030433

**Published:** 2022-03-19

**Authors:** Lisa Lardani, Giacomo Derchi, Vincenzo Marchio, Elisabetta Carli

**Affiliations:** Department of Surgical, Medical and Molecular Pathology and Critical Care Medicine, University of Pisa, 56121 Pisa, Italy; lisalardani@gmail.com (L.L.); gnolo78@gmail.com (G.D.); elisabettacarli1@gmail.com (E.C.)

**Keywords:** pediatric dentistry, primary teeth, bioactive materials, restorative dentistry, composite, direct restorations

## Abstract

Restorative procedures for caries affecting primary molars are a daily challenge for pediatric dentistry, and one of the main factors influencing the results of these restorative procedures is the choice of dental material used: bioactive materials were recently introduced, combining the strength of composites and the benefits of glass ionomers. The present study’s objective is to clinically evaluate the aesthetic, functional and biological properties of Activa™ Bioactive composite in approximal and occlusal carious lesions for 1 year using the FDI criteria for evaluating direct dental restorations. Forty-five children with occlusal or approximal caries in first or second primary molars were included in the study: the cavities were then randomized to be restored with either Activa BioActive or SDR Bulk-fill and evaluated over time according to Federation Dentaire Internationale (FDI) criteria. Results showed that Activa BioActive composite has similar performance over time compared to Bulk-fill composite, for both functional and aesthetic properties. Thus, within the limitations of this study, including the short follow-up period, it can be concluded that bioactive materials might be the material of choice to restore primary molars. A longer follow-up period is desirable to confirm these findings.

## 1. Introduction

Restorative procedures for decay affecting primary molars are a daily challenge for pediatric dentistry.

The prevalence of caries in the primary dentition varies between 60% and 90% worldwide [1], with percentages of 21.6% at 4 years of age and about 43.1% at 12 years of age in Italy [2].

Restorative treatment is part of a treatment plan that includes many factors: caries risk assessment, state of development of the dentition, the patient’s ability to cooperate [3], the compliance of parents to schedule post-treatment recalls and, last but not least, the choice of dental material used for the restoration. One of the main factors to consider in the treatment of the deciduous tooth is the compliance of the pediatric patient, a condition that often makes it difficult to isolate the field and compromises the adequate access to the lesion.

Conservative treatment aims to replace missing dental tissue to help control plaque, restore tooth function and protect the pulpo-dentinal complex by sealing the cavity produced after caries removal [4]. The ideal material for the restoration of deciduous teeth should have these properties: biocompatibility, bioactivity, aesthetics, handling, radiopacity and short processing times.

The restorative materials available for restoring posterior primary teeth include amalgam (AM), glass ionomer cement (GIC), resin-modified glass ionomer cement (RMGIC), high-viscosity glass ionomer cement (HVGIC), compomer (CP), composite resin (CR) and stainless-steel crowns (SSC) [3].

In dental literature, there is a lack of agreement regarding which of the various restorative materials is the best choice [5]; it has been reported that there is no sufficient scientific evidence in the literature to determine which is the best filling material for treating caries in the primary dentition [6].

There seems to be no evidence of superiority among restorative treatments using CP, GICs, AM and RC; thus, in clinical decision making, choosing one of these materials will depend on many different factors, including aesthetic requirements, using an easy and repeatable technique, caries activity, type of substrate and cavity design [7].

Resin composites and glass ionomers are two of the most used materials in pediatric restorative dentistry. When compared, resin composites show less porosity and surface roughness compared to glass ionomers, and significantly higher values of mechanical strength (334 ± 15.9 MPa and 78.8 ± 13.30 MPa for resin composites and glass ionomers, respectively).

Glass ionomers also have worse resistance to mechanical wear compared to resin composites [8] Glass ionomers, however, have the ability of releasing fluoride through time, and represent a cost-effective treatment ideal for pediatric dentistry [9].

Continued technological development in dental materials allowed the introduction of bioactive materials with characteristics similar to composites and glass ionomer cements, which can be especially useful in conservative and pediatric restorative dentistry.

These materials are called “bioactive” because they can activate dental tissue repair mechanisms, as well as eliciting a positive response from the dental tissue [10]. One of the critical factors in pediatric restorative dentistry is time, as children are often distracted and have varying levels of compliance: the use of a material with the ability to be placed in bigger increments without risking excessive polymerization shrinkage is needed. SDR Bulk-fill was chosen as a standard for comparison in this study because it is a flowable Bulk-fill composite: in the authors’ opinion, the ease of placement, adaptability, optimal mechanical properties and low polymerization shrinkage make this composite a choice material for the restoration of primary teeth. This Bulk-fill composite has minimal polymerization shrinkage (3.5%) and less polymerization stress with the same conversion rate of conventional composites [11].

Recently, a new bioactive restorative material called Activa™ BioActive-Restorative (Pulpdent Corp., Watertown, MA, USA) has been developed and introduced in the market.

This material aims to combine the strength and esthetics of composites with all the benefits of glass ionomers, mimicking the physical and chemical properties of natural teeth.

The components of Activa™ are a patented bioactive ionic resin, a patented rubberized resin, and a bioactive ionomer glass.

This material contains a matrix of bioactive ionic resin with a high release and recharge rate of calcium (Ca^2+^), phosphate (PO_4_^3−^) and fluoride (F^−^) ions; the rubberized resin is tough and durable, and contains reactive glass ionomer fillers that, in addition to a high fluoride release rate, mimic physical and chemical properties of natural teeth.

Activa BioActive composite was chosen as the experimental comparison to SDR Bulk-fill not only for its bioactive properties (capability of releasing fluoride) but also for its low polymerization shrinkage (1.7%) and high depth of light cure (4 mm), which allow for bigger increments and less time to complete the restoration [12].

Because of these properties, this material has many indications for class I and class II caries in primary molars.

According to the manufacturer, this material is also indicated in cases where the isolation is compromised or impossible and in patients with high caries index due to its fluoride-releasing properties.

However, the literature available on Activa™ is scarce.

SDR Bulk-fill and Activa BioActive composite were chosen for this trial because, compared to conventional composites and glass ionomer cements, they show properties which allow for easier clinical use combined with high mechanical resistance and bioactive proprieties (for Activa BioActive composite). The present study’s objective is to clinically evaluate the aesthetic, functional and biological properties of Activa™ BioActive composite in class II and I carious lesions for 1 year using the FDI criteria for evaluating direct dental restorations [13,14].

The FDI criteria set a background for the evaluation of dental restorations by introducing three groups of evaluation criteria: esthetic, functional and biological. Each of these groups has sub-groups with 16 evaluation criteria in total.

Regarding the aesthetic properties, four values were evaluated:

- Surface luster;
- Surface staining;
- Color stability and translucency;
- Anatomic form.

As for functional properties, seven values were evaluated:

- Retention and fracture of the restoration;
- Marginal adaptation;
- Wear of the restoration;
- Presence and persistence of the contact point;
- Presence of food impaction;
- Radiographic examination (if available);
- Patient’s view (opinion of the patient or, in this case, of the parent).

As for the biological properties, six values were evaluated:

- Post-operative sensitivity and tooth vitality;
- Secondary caries;
- Tooth integrity and enamel cracks;
- Periodontal response compared to a reference tooth;
- State of the mucosa adjacent to the restoration;
- Oral and general health.

For all three groups, five steps of grading were used for evaluation:

- 1: Clinically excellent/very good;
- 2: Clinically good;
- 3: Clinically sufficient/satisfactory;
- 4: Clinically unsatisfactory;
- 5: Clinically poor.

## 2. Materials and Methods

This blinded and split mouth design study was conducted at the University of Pisa, Italy.

Before treatment, children and their parents were informed about the procedure and signed an informed consent about the procedures and radiographical examinations needed.

All procedures were conducted in compliance with the Declaration of Helsinki and the study was approved by the ethics committee of Azienda Tutela Salute Sardegna (protocol 228/2020/CE, date of approval: 21 April 2020).

This study was carried out on 45 children who were admitted to the Department of Pediatric Dentistry at Santa Chiara University Hospital, Pisa (Italy).

Inclusion criteria were the following:

- Healthy children (ASA I score), both male and female, aged between 5 and 9 years, with a behavior rating of 3 or 4 according to the Frankl scale:
  ▪ Frankl 3: The child is cooperative, but somewhat reluctant/shy.
  ▪ Frankl 4: The child is completely cooperative and enjoys the experience) [15].
- All included children were required to have at least four carious vital primary molars, of which, at least one was on each side and on both jaws (according to the split mouth design of this study). These had to be class I or class II lesions according to Black’s classification and had to be present in the same type of tooth (e.g., upper and lower first primary molars, or upper and lower secondary molars). The caries had to be lower than an ICDAS score of five (distinct cavity with visible dentin) [16].
- All included teeth also had to be vital, restorable and without symptoms such as spontaneous pain, swelling, infection, fistulae, abscesses or tenderness on percussion.
- Radiographic inclusion criteria were the presence of radiolucency confined to the outer half of the dentine, normal Lamina Dura and periodontal space, and permanent teeth germ below the primary teeth in normal position with a predicted survival of the primary tooth of at least 2 years until normal exfoliation.
- Exclusion criteria were:
- Presence of systemic diseases.
- Teeth with discoloration, developmental defects or pathological mobility.
- Pulp exposure or indication for endodontic treatment or extraction.

In order to assure an adequate randomization between the two groups, one in which the Activa™ BioActive (Pulpdent Corporation, Watertown, MA, USA) composite resin was used and one in which the SDR Bulk-fill (Dentsply, Konstanz, Germany) composite resin was used, the procedure involved three operators and was applied as follows:

1. For each patient, cavities were numbered clockwise (1 to 4), starting from the first quadrant.
2. Local anesthesia was administered in order to prevent pain upon cavity preparation and maintain the child’s cooperation.
3. Involved teeth were isolated using a rubber dam (Hygienic Medium Fiesta Dam H04641, Coltene, Altstätten, Switzerland).
4. All cavities were cleaned and prepared by Operator 1 (OP1), a pediatric dentistry resident who had been previously trained to place both materials on deciduous teeth. OP1 used a high-speed diamond round bur (Intensiv FGM 200, Intensiv, Collina D’oro, Switzerland) and a round cutter in tungsten carbide (D + z CB7.314 010).
5. A wedge and metal matrix band (AutoMatrix, DentSply, Konstanz, Germany) were placed interproximally.
6. Selective enamel etching using a 35% orthophosphoric acid (Ultra-Etch, Ultradent Products, Inc., South Jordan, UT, USA) for 15 s was performed. Dentin was not etched [16].
7. A universal adhesive system compatible with deciduous teeth was applied according to the manufacturer’s instructions (Scotchbond™ Universal 3M ESPE, Maplewood, MN, USA).
8. Prior to the start of the appointment, Operator 2 (OP2) prepared (in a different room) a number of sealed opaque envelopes equal to the number of cavities ready to be restored, containing the indication to use either the Activa™ BioActive (Pulpdent Corporation, Watertown, MA, USA) composite resin or the SDR Bulk-fill (Dentsply, Konstanz, Germany) composite resin. Note that OP2 never entered the operative room and is blinded regarding the materials used for each cavity, since OP2 was involved in the evaluation procedure.
9. Operator 3 (OP3), after retrieving the sealed envelopes in the operative room, shuffled them in front of OP1 and let OP1 choose one. OP3 then opened the chosen envelope and instructed OP1 regarding the material to use to restore the tooth.
10. The restorative materials were polymerized following the instructions and the time of the production company.
11. The randomization procedure was repeated if there were more than two cavities; if not, the remaining envelope contained the indication for the last cavity remaining.
12. The congruity in the height of the restoration was verified using an articulation paper, and all the restorations were regularized with a diamond turbine cutter (Intensiv FGM 254 Collina D’oro, Switzerland) and then polished with the Venus Supra polishing kit (Kulzer Italia, Milano, MI, Italy).

**Figure 1:**
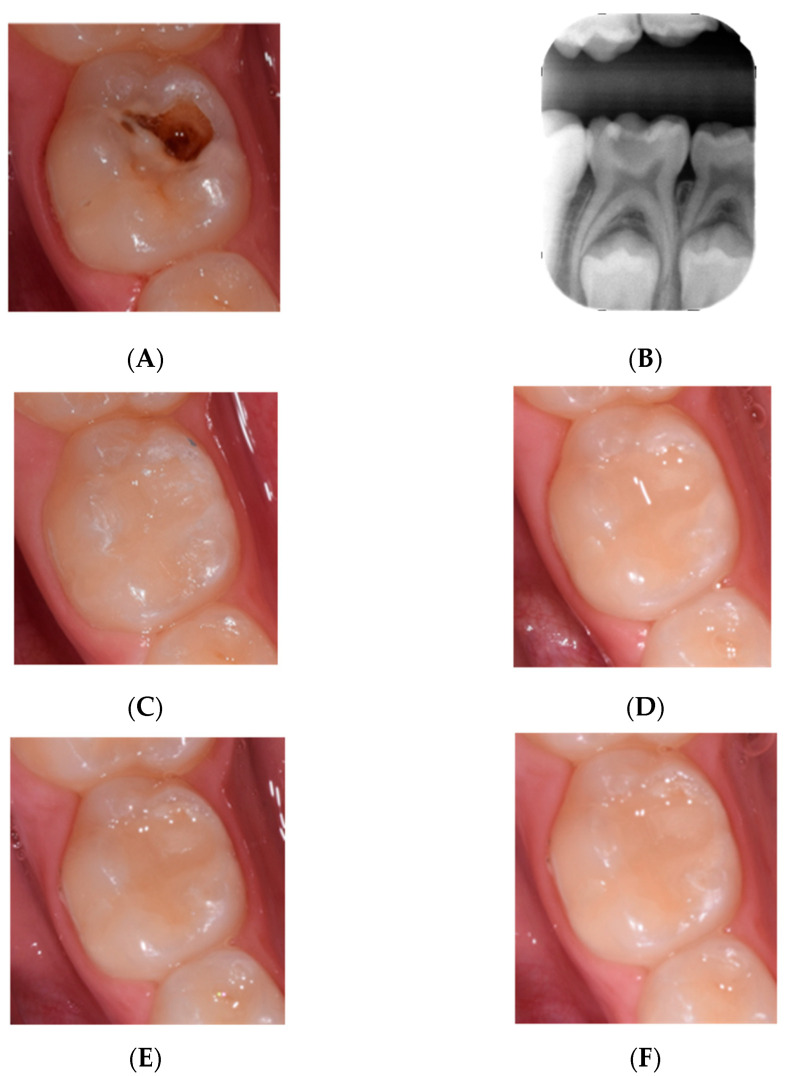
Example of a clinical case in which Activa BioActive was used. (**A**) pre operatory photograph, (**B**) pre operatory radiography, (**C**) post operatory, (**D**) 3 months post operatory, (**E**) 6 months post operatory and (**F**) 1 year follow up.

Following the operative appointment, the restorations were evaluated clinically and radiographically at 3, 6 and 12 months according to FDI-criteria [17]. All evaluations were carried out by OP2, which was not involved in the operative appointment. The esthetic aspects to be considered were assessed by direct observation in the absence of light provided by the unit lamp and, preferably, from a distance between 60 and 100 cm. Scores of 4 or 5 were considered as failure (Figure 1).

Teeth showing periapical problems at the radiographic evaluation within three months after restoration placement were excluded from the study because of the endodontic treatment needed. If there was a failure between follow-up appointments, further follow-up of restorative materials was terminated. If the tooth shed during the follow-up; however, treatment had to be considered successful even if follow-up had to be terminated.

Statistical analysis was carried out using MatLab software (MatLab, Natick, MA, USA).

For the statistical analysis of the data concerning the aesthetic, functional and biological properties were used for univariate analysis, which evaluated:

Kruskal–Wallis nonparametric ANOVA test was used for testing the behavior over time of the two materials at T0, T1, T2, T3 and in the two respective types of classes (class I and class II)

Chi-square analysis was used for testing the behavior of the material at each time points (T0, T1, T2 and T3) comparing the “excellent” (score = 1) and “not excellent” scores (score > 1) between the two types of material with a level of significance equal to *p* < 0.05 (significant difference) and *p* < 0.01 (highly significant difference).

A bivariate analysis was subsequently used by evaluating the combined effectiveness of time and material tested on the number of samples excellent, in all samples and separately in class I and class II samples.

## 3. Results

A total of 45 children, 28 girls (62.2%) and 17 boys (37.8%), aged between 5 and 9 years old (mean 6.15 ± 0.98 years) were included in this study, with a total of 180 primary molars presenting class II and class I lesions (81 primary first molar, 98 primary second molar), that required treatment (Table 1).

At the end of the trial, no dropouts were reported, and all patients were present in all follow-ups (dropout rate: 0%). Only one patient was excluded upon treatment because endodontic treatment of the involved teeth became necessary.

The restorations with the bioactive composite, Activa BioActive, were performed on 90 deciduous teeth: 34 were class I restorations and 56 were class II restorations.

The restorations with SDR were performed on 89 deciduous teeth: 33 were class I restorations and 56 were class II restorations (Table 2).

The overall failure rates at 12 months were 2.2% (2) and 2.2% (2) for the two groups, respectively.

The failures were due to total restorations loss in class II restorations and were 1 at T2 (6 months) and 1 at T3 (12 months) for the teeth restored with bioactive composite Activa BioActive.

The failures for the restorations made with the Bulk-fill composite were 1 at T2 and 1 at T3, both class II restorations (Table 3).

### 3.1. Aesthetic Properties

The aesthetic properties survey for the bioactive composite Activa BioActive provided the following results (Table 4):

- At T0, there were 90 “clinically excellent” restorations (100%).
- At T1, there were 80 “excellent” restorations (89%) and 10 “good” restorations (11%).
- At T2, there were 65 “clinically excellent” restorations (72.2%), 23 “clinically good” restorations (25.5%), 1 “clinically sufficient” restoration (1.1%) and 1 failure (1.1%).
- At T3, there were 60 “clinically excellent” restorations (67%), 27 “clinically good” restorations (30%), 1 “clinically sufficient” restoration (1%) and 2 failures (2%).

The aesthetic properties of the restorations made with the Bulk-fill composite were analyzed at time points (Table 4):

- At T0, there were 89 “clinically excellent” restorations (100%).
- At T1, there 73 restorations defined as “clinically excellent” (82%) and 16 “clinically good” restorations (18%).
- At T2, there were 57 “clinically excellent” restorations (64%) and 31 “clinically good” restorations (35%) and 1 failure.
- At T3, there were 53 “clinically excellent” restorations (60%) and 33 “clinically good” restorations (37%) and 1 “clinically sufficient” (1%) and 2 failures (2%).

The Kruskal–Wallis test determined that there was a statistically significant (K-W *p* < 0.001) decrease over time in the number of restorations with Activa BioActive defined as “clinically excellent”, up to 33% at 1 year (T3).

Bulk-fill composite restorations also saw a statistically significant (K-W *p* < 0.001) in the number of restorations defined as “clinically excellent” from the aesthetic point of view to 40% of the total at 1 year (T3) (Table 5).

This behavior is mainly related to class II restorations which, as seen in Table 6, have shown a decrease in the number of “clinically excellent” restorations over time: at T0, there were 56; at T1, there were 46; at T2, there were 31; and finally, at T3, there were 27.

The number of restorations defined as “clinically not excellent”; therefore, those with a score greater than 1 were: 0 restorations at T0, 10 restorations a T1, 25 restorations at T2 and 29 restorations at T3.

Class I restorations did not show the same behavior in aesthetic properties at various time points, instead maintaining the number of “excellent” restorations unchanged through time, except at 1 year (T3) where only one restoration was defined as “not excellent”; no failures were registered.

Similar to the restorations made with the Bioactive materials, the decrease in the number of “clinically excellent” restorations in the SDR group is ascribable to the class II restorations. At T0, 56 restorations were defined as “clinically excellent”, at T1 there were 40, at T2 here were 24 and at T3 only 20 restorations, while the class I restorations did not show the same decrease over time and their behavior regarding aesthetic properties remained unchanged over time: 100% of class I restorations were defined “clinically excellent” throughout the various time points.

By comparing the two materials at different time points (T0, T1, T2 and T3), it was observed that at T0, there was no significant difference in the aesthetic properties between Bioactive composite restorations and Bulk-fill composite restorations (*p* = 1); at T1, there was no statistically significant difference (*p* = 0.19), as at T2 (*p* = 0.24) and at T3 (*p* = 0.32).

Both groups showed a decrease in “clinically excellent” restorations over time, but a significant difference in the behavior of the two materials on the number of excellent samples over time was reported (*p* = 0.045).

By comparing the behavior of the two materials based on the restorations’ class, it can be hypothesized that, in terms of esthetic properties:

- Regarding both class I and class II restorations, there was no significant difference at different time points between the two materials; therefore, it can be suggested that there is no influence of the type of restoration on the esthetic result of the materials over time.
- There was no significant difference in class II restorations over time at the various time points (*p* = 0.083).

### 3.2. Functional Properties

The functional properties survey for Activa BioActive provided the following results:

- At T0, there were 90 “clinically excellent” restorations (100%)
- At T1, there were 84 “clinically excellent” restorations (93%) and 6 “clinically good” restorations (7%)
- At T2, there were 76 “clinically excellent” restorations (84%) and 12 “clinically good” restorations (13%), 1 “clinically insufficient but repairable” restoration and 1 failure.
- At T3, there were 75 “clinically excellent” restorations (83%), 11 “clinically good” restorations (12%), 2 “clinically sufficient” restorations (2%) and 2 failures (2%).

The functional properties for Bulk-fill composite show a trend very similar to Activa BioActive restorations with the Bioactive composite:

- At T0, there were 89 “clinically excellent” restorations (100%).
- At T1, there were 85 “clinically excellent” restorations (96%) and 4 “clinically good” restorations (4%).
- At T2, there were 79 “clinically excellent” restorations (89%), 9 “clinically good” restorations (10%) and 1 failure.
- At T3, there were 69 “clinically excellent” restorations (78%), 18 “clinically good” restorations (20%) and 2 failures (2%).

The functional properties of Bioactive composite restorations, much like the esthetic properties, saw a change over time with a decrease in restorations defined as “clinically excellent”. However, this difference over time was not significant (Table 7).

As seen in Table 8, for Activa BioActive, class I restorations maintained a “clinically excellent” evaluation throughout the trial (T0 to T3). Class II restorations with a “clinically excellent” evaluation were 56 at T0, 50 at T1, 42 at T2 and 41 at T3; the restorations with a “clinically not excellent” evaluation and score higher than 1 were 0 at T0, 6 at T1, 14 at T2 and 15 to T3.

For SDR Bulk-fill restorations, a similar trend was observed in class II restorations, but not in class I restorations, as previously reported for aesthetic properties.

A decrease of “clinically excellent” Bulk-fill composites in terms of functional properties has been detected; however, this difference over time is not statistically significant.

There is also no statistically significant difference (*p* > 0.005) at different time points in the number of restorations defined as “clinically excellent”.

The behavior of class I and class II Bulk-fill composite restorations is the same as the behavior of Bioactive composite restorations.

In fact, the “clinically excellent” class I restorations were 33 at all time points; the “clinically excellent” class II restorations were 56 at T0, 52 at T1, 46 at T2 and 36 at T3. The “clinically not excellent” restorations with a score higher than 1 were 0 at T0, 4 at T1, 10 at T2 and 20 at T3.

A tendency towards a decrease in the number of restorations assessed as “clinically excellent” only in class II restorations, which is not observable in class I restoration, has been observed.

Comparing the two materials at different time points, it has been observed that there is no statistically significant difference in functional properties between restorations made with BioActive composite and Bulk-fill composite at T0 (*p* = 1) at T1 (*p* = 0.53) at T2 (*p* = 0.38) and at T3 (*p* = 0.31).

Comparing the behavior of the two materials based on the class type it has been observed that there is no statistically significant difference between the number of “Clinically excellent” restorations (*p* = 1) and that there is no statistically significant difference between the two materials for class I and class II restorations (*p* = 0.56).

### 3.3. Biological Properties

Regarding the biological properties (Table 9), it has been observed that, for the restorations made with the Bioactive composite Activa BioActive:

- At T0, there were 90 “clinically excellent” restorations (100% restorations),
- At T1, there were 88 “clinically excellent” restorations (98%) and 2 “clinically good” restorations (2%).
- At T2, there were 88 “clinically excellent” restorations (98%) and 1 “clinically sufficient” restorations (1%).
- At T3, there were 87 “clinically excellent” restorations (97%) and 1 “clinically sufficient” restoration (1%) and 2 failure (2%).

There was accordingly a decrease over time in the number of restorations defined as “clinically excellent”, but it was not statistically significant.

Regarding the biological properties of the restorations made with Bulk-fill composite, the results are very similar to those of the BioActive composite:

- At T0, the restorations defined as “clinically excellent” were 89 (100%).
- At T1, they were 88 (99%) and 1 (1%) “clinically good”.
- At T2, the “clinically excellent” were 88 (99%).
- At T3, the “clinically good” restorations were 1 (1%) and the excellent ones 86 (97%) and there were also 2 (2%) failures.

As seen in Table 10, the biological properties of class I and II restorations with BioActive composite had the same trend: class I restorations defined as “clinically excellent” were 34 and remained unchanged for all time points.

For class II restoration, a decrease in “clinically excellent” restorations was observed: there were 56 clinically excellent restorations at T0, 54 at T1, 54 at T2 and 53 at T3.

Furthermore, the class I and class II restorations made with Bulk-fill composite showed a similar trend and the restorations defined as “clinically excellent” remained, in the case of the class I restorations, unchanged over time; in fact there were 33 “clinically excellent” restorations throughout all the time points, while for class II restorations there were 56 “clinically excellent” restorations at T0, 55 at T1 and T2 (with 2 “acceptable” restorations) and 53 at T3 (with 3 “acceptable” restorations).

Comparing the two materials at time points T0, T1, T2 and T3, it was observed that there was no significant difference in biological properties between restorations made with BioActive composite and those made with Bulk-fill composite at T0 (*p* = 1), T1 (*p* = 0.57), T2 (*p* = 0.32) and T3 (*p* = 0.99).

Considering both material type and cavity class, the behavior over time showed no significant difference in biological properties (*p* = 0.16). There was also no significant difference between class I restorations and class II restorations (*p* = 0.16).

## 4. Discussion

Direct restorations in primary teeth are not as simple as it might seem: the mesial (or distal) marginal ridge of a primary tooth has a shorter occluso–cervical dimension, with a much more concave aspect, which makes it difficult to obtain an ideal marginal seal.

In addition, primary teeth have interproximal contact surfaces instead of interproximal contact points, which are characteristic of secondary teeth: this makes it even more difficult to obtain an ideal restoration, which prevents food impaction from happening. As already discussed, in addition to anatomical challenges in restoring primary teeth, the time factor and short attention span of children from 5 to 9 years of age adds to an already complex situation, making class II restorations of primary teeth complex restorations that need specific formation and ideal materials to work quickly and effectively.

The present study compared two adhesive materials: Activa BioActive and SDR Bulk-fill, which were used for the restoration of primary molars and compared their aesthetic, functional and biological properties over time, also evaluating the same properties from the point of view of cavity class (class I or class II restorations).

Regarding aesthetic properties, the two materials had a similar behavior with a worsening of the values at the various time points; in particular, the esthetic properties of restorations made with Activa BioActive worsened at T3 by 33%, while for restorations made with Bulk-fill SDR, worsened by 40% at the same time point. In class I, the two materials behave similarly, while in Class II there seems to be a tendency toward a statistically significant difference, favoring the Activa BioActive composite. This worsening of esthetic properties is prominent for class II restorations: this can be explained by the fact that the cavity design of class II restorations is more complex compared to class I restorations, and the restoration itself is technically more challenging; in particular, the amount of material applied, the longer time needed and the difficulty in cleaning the interproximal surfaces are all factors that influence this kind of restoration in a negative way. Additionally, class II restorations undergo greater forces and stresses during chewing. Longer follow-up times are needed to further investigate this difference.

The functional properties of both Bioactive composite restorations and restorations with Bulk-fill composites changed over time with a decrease in restorations defined as “clinically excellent”. This difference in time was not significant, probably because the follow-up time considered was short, while most of the studies on deciduous teeth in the literature evaluated the follow-up for at least two years. Comparing the restorations based on the restoration class, the “clinically excellent” values in class I restorations remained unchanged over time; for class II restoration, as already observed for the esthetic values, the restorations defined as “clinically excellent” tended to decrease but not in a statistically significant manner. Thus, it can be concluded that for functional properties, there seems to be no difference between the two materials, even when compared based on restoration class: a longer follow-up time, however, may be needed to confirm this finding.

The biological properties of Bioactive composite and Bulk-fill composite restorations changed over time, with a slight decrease in restorations defined as “clinically excellent”, but the difference between materials was not significant even when the restoration class was considered.

The overall failure rate at T3 was 2% and was the same for the two materials; the Activa composite BioActive and the Bulk-fill composite, consequently, seem to be comparable.

This study has some shortcomings that need to be addressed: a longer follow-up time is needed to further highlight differences already observed in this study, and in order to evaluate the properties of class II restorations thoroughly, a greater number of samples is needed; the fact that the restorations were performed by a single operator prevented the authors from considering the influence of the operator’s skill on the properties examined. Future objectives are to design a broader study and to include an evaluation of the operator’s experience and skill influence on these properties over time, and to observe the exfoliated deciduous teeth through lab analyses in order to evaluate the seal quality on class II restorations’ margins.

## 5. Conclusions

The data from this study indicate that the Activa BioActive bioactive composite had the same performance of Bulk-fill composite after a one-year follow-up.

These results encourage us to use bioactive materials also in primary teeth, exploiting the multiple properties they provide, in particular, the ion exchange in acidic conditions which was an exclusive characteristic of glass ionomer cements. These materials can also be used in conditions of difficult isolation and especially in children where collaboration is not adequate, due to multiple polymerization mechanisms available in the same material.

Long-term data, however, will be needed to allow the evaluation of the deciduous element to be restored until its exfoliation to observe the possible presence of hydroxyapatite at the interface between dental tissue and material, a characteristic of bioactive materials, as indicated by the manufacturer.

A longer follow-up will also allow to evaluate whether the properties of the two materials over time are similar or not.

Based on this study’s findings, Activa BioActive-Restorative, by combining the characteristics and advantages of cements glass ionomers and composites, might be a material of choice to restore a primary molar.

However, it is important to remember that the success of conservative treatment cannot and should not be linked only to the type of material chosen but it is linked, above all, to the change in lifestyle of the child and the family, so prevention remains the key to success in conservative dentistry.

## Figures and Tables

**Table 1:** Age and gender distribution.

	Average	Standard Deviation (SD)
Age	6.15	0.98
	Male	Female
Gender	17	28

**Table 2:** Involved teeth and class restoration distribution.

Activa BioActive		SDR Bulk-Fill	
Class I	Class II	Class I	Class II
34	56	33	56
First Primary Molars	Second Primary Molars	First Primary Molars	Second Primary Molars
41	49	40	49

**Table 3:** Restoration failures.

	Failures, *n* (%)				
	T0	T1	T2	T3	T4
Activa BioActive	0 (0)	0 (0)	1 (1, 1)	2 (2, 2)	2 (2, 2)
SDR Bulk-fill	0 (0)	0 (0)	1 (1, 1)	2 (2, 2)	2 (2, 2)

**Table 4:** Aesthetic properties evaluation based on FDI criteria.

	Aesthetic Properties							
	Activa BioActive				SDR Bulk-Fill			
	*n* (%)				*n* (%)			
FDI CRITERIA	T0	T1	T2	T3	T0	T1	T2	T3
1—CLINICALLY EXCELLENT	90 (100)	80 (89)	65 (72)	60 (67)	89 (100)	73 (82)	57 (64)	53 (60)
2—CLINICALLY GOOD	0 (0)	10 (11)	23 (26)	27 (30)	0 (0)	16 (18)	31 (35)	33 (37)
3—CLINICALLY SUFFICIENT	0 (0)	0 (0)	1 (1)	1 (1)	0 (0)	0 (0)	0 (0)	1 (1)
4—CLINICALLY UNSATISFACTORY	0 (0)	0 (0)	0 (0)	0 (0)	0 (0)	0 (0)	0 (0)	0 (0)
5—CLINICALLY POOR	0 (0)	0 (0)	0 (0)	0 (0)	0 (0)	0 (0)	0 (0)	0 (0)
FAILED	0 (0)	0 (0)	1 (1)	2 (2)	0 (0)	0 (0)	1 (1)	2 (2)

**Table 5:** Overall evaluation of aesthetic properties of restorations based on material used.

	Aesthetic Properties							
	Activa BioActive *n* (%)				SDR Bulk-Fill *n* (%)			
	T0	T1	T2	T3	T0	T1	T2	T3
Excellent	90 (100)	80 (89)	65 (72)	60 (67)	89 (100)	73 (82)	57 (64)	53 (60)
Non-Excellent	0 (0)	10 (11)	25 (28)	30 (33)	0 (0)	16 (18)	32 (36)	36 (40)

**Table 6:** Overall evaluation of aesthetic properties based on material used and restoration class.

	Aesthetic Properties															
	Activa BioActive *n* (%)								SDR Bulk-Fill *n* (%)							
	Class I				Class II				Class I				Class II			
	T0	T1	T2	T3	T0	T1	T2	T3	T0	T1	T2	T3	T0	T1	T2	T3
Excellent	34	34	34	33	56	46	31	27	33	33	33	33	56	40	24	20
Non-Excellent	0	0	0	1	0	10	25	29	0	0	0	0	0	16	32	36

**Table 7:** Functional properties evaluation based on FDI criteria.

	Functional Properties							
	Activa BioActive				SDR Bulk-Fill			
	*n* (%)				*n* (%)			
FDI CRITERIA	T0	T1	T2	T3	T0	T1	T2	T3
1—CLINICALLY EXCELLENT	90 (100)	84 (93)	76 (84)	75 (83)	89 (100)	85 (96)	79 (89)	69 (78)
2—CLINICALLY GOOD	0 (0)	6 (7)	12 (13)	11 (12)	0 (0)	4 (4)	9 (10)	18 (20)
3—CLINICALLY SUFFICIENT	0 (0)	0 (0)	0 (0)	2 (2)	0 (0)	0 (0)	0 (0)	0 (0)
4—CLINICALLY UNSATISFACTORY	0 (0)	0 (0)	1 (1)	0 (0)	0 (0)	0 (0)	0 (0)	0 (0)
5—CLINICALLY POOR	0 (0)	0 (0)	0 (0)	0 (0)	0 (0)	0 (0)	0 (0)	0 (0)
FAILED	0 (0)	0 (0)	1 (1)	2 (2)	0 (0)	0 (0)	1 (1)	2 (2)

**Table 8:** Overall evaluation of functional properties based on material used and restoration class.

	Functional Properties															
	Activa BioActive *n* (%)								SDR Bulk-Fill *n* (%)							
	Class I				Class II				Class I				Class II			
	T0	T1	T2	T3	T0	T1	T2	T3	T0	T1	T2	T3	T0	T1	T2	T3
Excellent	34	34	34	34	56	50	42	41	33	33	33	33	56	52	46	36
Non-Excellent	0	0	0	0	0	6	14	15	0	0	0	0	0	4	10	20

**Table 9:** Biological properties evaluation based on FDI criteria.

	Biological Properties							
	Activa BioActive				SDR Bulk-Fill			
	*n* (%)				*n* (%)			
FDI CRITERIA	T0	T1	T2	T3	T0	T1	T2	T3
1—CLINICALLY EXCELLENT	90 (100)	88 (98)	88 (98)	87 (97)	89 (100)	88 (99)	88 (99)	86 (97)
2—CLINICALLY GOOD	0 (0)	2 (2)	0 (0)	0 (0)	0 (0)	1 (1)	0 (0)	1 (1)
3—CLINICALLY SUFFICIENT	0 (0)	0 (0)	1 (1)	1 (1)	0 (0)	0 (0)	0 (0)	0 (0)
4—CLINICALLY UNSATISFACTORY	0 (0)	0 (0)	0 (0)	0 (0)	0 (0)	0 (0)	0 (0)	0 (0)
5—CLINICALLY POOR	0 (0)	0 (0)	0 (0)	0 (0)	0 (0)	0 (0)	0 (0)	0 (0)
FAILED	0 (0)	0 (0)	0 (0)	2 (2)	0 (0)	0 (0)	0 (0)	2 (2)

**Table 10:** Overall evaluation of Functional properties based on material used and restoration class.

	Biological Properties															
	Activa BioActive *n* (%)								SDR Bulk-Fill *n* (%)							
	Class I				Class II				Class I				Class II			
	T0	T1	T2	T3	T0	T1	T2	T3	T0	T1	T2	T3	T0	T1	T2	T3
Excellent	34	34	34	34	56	54	54	53	33	33	33	33	56	55	55	53
Non-Excellent	0	0	0	0	0	2	2	3	0	0	0	0	0	1	1	3

## Data Availability

The data supporting the results have already been included in the study “Results” sections. Patient-specific data are not available due to privacy regulations.
